# Traditional vs. Energetic and Perchlorate vs. “Green”: A Comparative Study of the Choice of Binders and Oxidising Agents

**DOI:** 10.3390/molecules28155787

**Published:** 2023-07-31

**Authors:** Kinga Lysien, Sylwia Waśkiewicz, Agnieszka Stolarczyk, Anna Mielańczyk, Roman Zakusylo, Tomasz Jarosz

**Affiliations:** 1 Department of Physical Chemistry and Technology of Polymers, Silesian University of Technology, 44-100 Gliwice, Poland; 2 Shostka Institute, Sumy State University, 41100 Shostka, Ukraine

**Keywords:** solid propellant, perchlorate, hydroxyl-terminated polybutadiene, glycidyl azide polymer green, oxidising agent, phase-stabilised ammonium nitrate, DSC

## Abstract

The aim of this article is to compare rocket propellants containing a traditional binder (hydroxyl-terminated polybutadiene) and an energetic binder (glycidyl azide polymer), as well as a perchlorate oxidising agent and a “green” one, i.e., ammonium perchlorate and phase-stabilised ammonium nitrate. We have outlined the effects of individual substances on the sensitivity parameters and decomposition temperature of the produced solid propellants. The linear combustion velocity was determined using electrical methods. Heats of combustion for the propellant samples and the thermal decomposition features of the utilised binders were investigated via differential scanning calorimetry (DSC). Activation energy values for the energetic decomposition of the propellants were determined via the Kissinger method, based on DSC measurements at varied heating rates.

## 1. Introduction

Solid propellants have found a wide range of civilian and military applications, both in large- and micro-scale propulsion systems [1]. The main components of solid propellants are the oxidising agent and binder, which also fulfils the role of fuel. These components are typically supplemented by auxiliary components, such as metallic fuels, combustion modifiers, plasticisers, and other additives [2].

Many of the solid propellants reported in the recent literature utilise hydroxyl-terminated polybutadiene (HTPB) as the binder [2]. This stems from the fact that HTPB exhibits a range of favourable properties, including good adhesive properties, high heat of combustion, and high stability [3,4]. It should be noted, however, that HTPB requires curing, which is typically conducted using highly toxic isocyanates, and exhibits a very strongly negative oxygen balance value (e.g., −313 % for HTPB cured by toluene diisocyanate) [5]. This translates to the need for HTPB-based solid propellants to contain a significant amount of oxidising agent, achieving a high degree of loading of the polymer with solids.

The above does not preclude producing viable fuels, as seen by the popularity of the ammonium perchlorate (AP)-based AP/HTPB propellant base, but limits the extent to which other auxiliary compounds can be utilised. This, in turn, limits the achievable parameters of the propellants. Consequently, alternative materials were sought after, leading to the development of “energetic binders” (EBs). Most of the early attempts at EBs involved equipping polymer chains with explosophore groups, such as nitrate (-ONO_2_), nitro (-NO_2_), and nitramino (-NHNO_2_) and azido (-N_3_) functionalities [6]. Later on, research on EBs focused on polymers bearing oxygen atoms in their main chain (e.g., polyoxetanes, polyoxiranes) rather than hydrocarbon main chains [7,8,9]. Currently, the benefits of using EBs are widely recognised, even though solid propellant formulations based on “traditional” binders, such as HTPB, remain in common use [10].

In regards to the oxidising agent utilised in solid propellant formulations, AP is among the most popular, as its affords the resultant propellants high specific impulses and high reliability [11,12]. It is also possible to fine-tune both the porosity and particle size of AP [13], as well as to modify its reactivity by supplementing it with catalysts, typically metal oxides [14,15]. The main drawback of AP is that its combustion results in the formation of hydrogen chloride, chlorine, and chlorine oxides, which are highly problematic atmosphere pollutants [16,17]. Consequently, significant research interest is devoted to developing oxidising agents for solid propellants that can replace AP.

Potential alternatives to AP include trinitroethyl esters [18], ammonium dinitramide [19], and nitrates (e.g., ammonium nitrate) [20,21,22]. The former two oxidising agents are highly reactive and can yield propellants exhibiting favourable parameters, but require complex synthesis and are relatively expensive. Nitrates and ammonium nitrate, in particular, are relatively inexpensive, but due to their low reactivity, produce propellants with limited parameters. Consequently, the application of nitrates in solid propellants typically requires the use of catalysts to achieve reasonable performance [23,24].

In this work, we have presented our studies on model solid propellants, comparing the use of “traditional” and energetic binders, as well as the use of AP and phase-stabilised ammonium nitrate as the oxidising agents.

## 2. Results and Discussion

### 2.1. Friction and Impact Sensitivity

Regardless of the oxidising agent used (Table 9), samples containing glycidyl azide polymer (GAP) as a binder were more sensitive to friction (Table 1) than those where HTPB was used as the binder. This stems from the fact that GAP has a more rigid polymer chain than HTPB, due to the presence of azido groups [6], translating to a higher hardness upon curing. As expected, samples containing ammonium perchlorate showed higher friction sensitivity, compared to samples containing phase-stabilised ammonium nitrate (PSAN).

Interestingly, the impact sensitivity of the samples is presented quite differently from that of friction sensitivity. Regardless of the oxidising agent used, samples containing HTPB are more sensitive to impact. This may be an issue related to the utilised standard methodology, in which the impact of the fallhammer produces adiabatic compression of air in the sample chamber, as has been recently pointed out in literature [25]. This compression may be enough to induce decomposition of the samples, thereby influencing the result of the test for the investigated samples, particularly in light of the solid rocket propellant (SRP) samples containing both magnesium dust and nitroguanidine (NQ). This feature may also be a result of the higher hardness of GAP-bearing samples, in which more of the released mechanical energy is consumed for fracturing the cured polymer matrix than in the case of HTPB-bearing samples.

### 2.2. Determination of Ignition/Explosion Temperature

The results of the determination of the ignition/explosion temperature of the samples are shown in Table 2. As in the case of the determination of sensitivity to friction, samples containing GAP have a lower ignition temperature—it can be concluded that samples containing this polymer have a higher sensitivity to flame and temperature than samples containing HTPB. Again, the effect of the oxidising agent used was observed—the high-energy decomposition process for PSAN-containing samples can occur at a lower temperature than for AP-containing samples.

### 2.3. Determination of Linear Combustion Velocity

The results of combustion velocity tests are presented in Table 3. A higher burning rate was recorded for samples containing GAP as a binder. Again, such a result can be linked to the presence of additional high-energy azide groups in the polymer. Comparing the effect of the oxidising agent used on the combustion velocity obtained, it can be seen that a higher value of linear combustion velocity was obtained for samples with AP. This is due to the fact that ammonium perchlorate is a more reactive and, therefore, more energetic oxidising agent.

### 2.4. Study of the Kinetics of High-Energy Transformations

**Figure A1:**
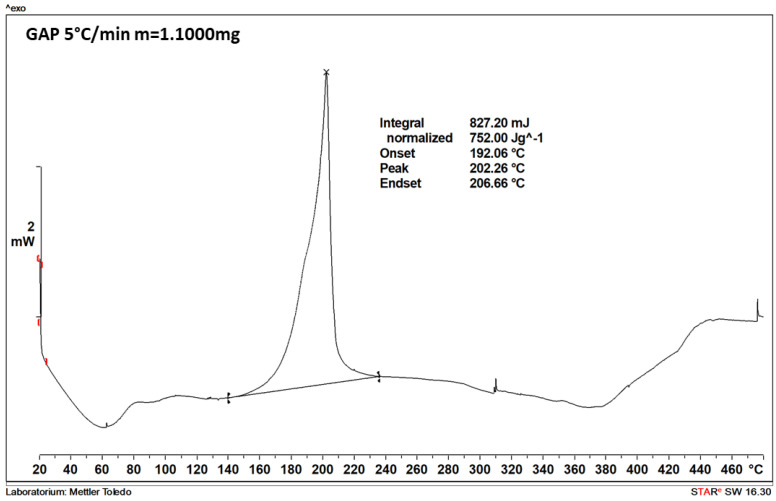
The DSC thermogram recorded for GAP (sample mass of 1.1 mg).

**Figure A2:**
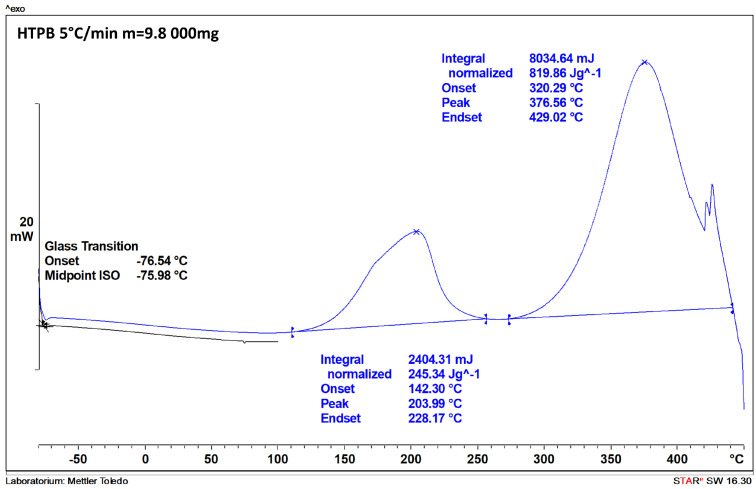
The DSC thermogram recorded for HTPB (sample mass of 9.8 mg).

The DSC thermograms of the polymers used as the binders (Figure A1 and Figure A2) are consistent with those reported in the literature, with their features being summarised in Table 4. In the case of HTPB, the first exothermic peak can be attributed to the cis-trans isomerisation, cyclisation, and cross- linking of HTPB [26]. The second peak, in turn, is associated with the decomposition of the polymer, as in the case of the first exothermic peak observed for GAP [27].

The DSC studies of the SRP samples were conducted at multiple heating rates, i.e., 3 K/min, 5 K/min, 7 K/min, 10 K/min, 12 K/min, and 15 K/min, so as to allow the determination of the activation energy values for the reactions taking place in the samples (Table 5, Table 6 and Table 7). In the case of SRP-1 (containing HTPB and PSAN), the results of the DSC measurements were considered to not be sufficiently reliable. Despite additional standardisation measures being taken, the DSC thermograms showed poor repeatability. These issues may be linked to interactions of the ZnO and CuO in PSAN with HTPB or to minor impurities in the obtained polymer.

In terms of the heat of combustion of the SRP samples, we have chosen the lowest employed heating rate (i.e., 3 K/min) as the diagnostic one, due to the fact that it introduces the least thermal inertia. When the heats of combustion of the samples are arranged in descending order, the sequence SRP-2 > SRP-1 > SRP-3 is established. This is in line with expectations, due to HTPB containing a much higher carbon atom content than GAP.

An interesting observation is the comparison of the ignition temperatures of the SRP samples (Table 2) with the temperatures of the exothermic peak temperatures of the binders—the use of GAP, which shows a peak at temperatures approximately 40 °C higher than HTPB translates to an increase in SRP ignition temperature, respectively, by 16 °C (SRP-2 vs SRP-1) and by 30 °C (SRP-4 vs SRP-3), depending on the utilised oxidising agent.

The results of the E_a_ investigation are presented in Table 8. The sample containing GAP and PSAN exhibits the lowest activation energy, whereas the sample containing HTPB and AP exhibits the highest one. From the point of view of rocket propellants, it is more favourable to obtain lower activation energies, as this indicates that high-energy decomposition reactions occur more easily.

## 3. Materials and Methods

### 3.1. Materials

The materials used in this work, along with their suppliers and any relevant notes are listed in Table 9. It should be noted that glycidyl azide polymer (GAP) and nitroguanidine (NQ) have been synthesised for the purpose of this work, as per the indicated procedures published in literature and described in the following sections.

### 3.2. Synthesis

GAP was synthesised according to the literature [29]. The first step was the polymerisation of epichlorohydrin using ethylene glycol, boron trifluoride-diethyl ether adduct (BF_3_·Et_2_O) as catalyst and dichloromethane as a solvent. The next step was the substitution of chloride groups with azide groups via a reaction with sodium azide.

Nitroguanidine was prepared according to [30]. Nitroguanidine was obtained by reacting concentrated sulphuric acid with guanidine nitrate. The precipitate obtained in this reaction was then recrystallised from deionised water.

### 3.3. NMR and IR Spectra of Synthesised Substances

^1^H-NMR analysis was performed for solutions in CDCl_3_ on a Varian Unity Inova (Palo Alto, CA, USA) spectrometer with a resonance frequency of 300 MHz using TMS as the internal standard. IR spectroscopy was carried out on a PerkinElmer Spectrum Two (Waltham, MA, USA) spectrometer with a UATR (Single Reflection Diamond (Waltham, MA, USA)) module. The results of spectroscopic investigations for the synthesised samples are summarised in Table 10 and Table 11.

In the case of GAP, the most characteristic peak occurs in the range of about (3.00–3.80 ppm)—a peak originating from protons of the -CH_2_N_3_ group. It is impossible to accurately determine the value of the shift characteristic of this group, due to the overlapping signals from the other groups present in the repeat unit.

For glycidyl azide polymer, the IR spectrum contains signals at 2101 cm^−1^ and at 1258 cm^−1^, originating from stretching vibrations of the azide group. The absorption band present at 1089 cm^−1^ corresponds to C-O-C stretching vibrations from the ether chain connecting the moieties in the polymer molecule.

Absorption bands, characteristic of -NO_2_ group vibrations, are observed for nitroguanidine at 1394 cm^−1^ and at 1631 cm^−1^. Absorption bands at 3259 cm^−1^ and at 339 cm^−1^ correspond to vibrations originating from the N-H bond.

### 3.4. Preparation of Solid Propellant Samples

Rocket propellant samples were prepared in 50 mL glass beakers. Mixing was performed by hand with a glass stirring rod used. The cross-linking process was carried out at 60 °C. The exact composition of the produced solid propellant samples is shown in Table 12.

High hygroscopicity is a major disadvantage of ammonium nitrate (AN), as the absorption of water by rocket propellants significantly decreases their performance. In order to assess whether the use of phase-stabilised ammonium nitrate (PSAN) can mitigate this issue to some extent, gravimmetry was employed. The investigated samples of AN and PSAN of similar mass were left in identical open containers in a climate chamber maintaining a temperature of 25 °C and a relative humidity of 33 %. The samples were periodically taken out of the chamber and weighed to determine the amount of moisture absorbed by the two oxidising agents over time. A comparison of the results of the mass change measurements reveal that, although initially PSAN absorbs almost as much moisture as AN (0.014 % vs. 0.019 % after 30 min), after 24 h (1440 min) have elapsed, its absorption of moisture is approximately 50 % less than in the case of AN (Table 13). Consequently, though the inherent hygroscopicity of AN cannot be negated, its use in the form of PSAN mitigates this issue to a significant extent.

### 3.5. Investigation of Sensitivity to Impact and Friction

Friction and impact sensitivity values were determined according to the relevant international standards [31,32] using a Peters Friction Apparatus and a BAM Fallhammer, respectively.

### 3.6. Determination of Ignition/Explosion Temperature

The ignition/explosion temperatures of the produced solid propellants were determined using an Automatic Explosion Temperature 402 Tester (OZM Research, Bliznovice, Czech Republic). The ignition/explosion temperature measurement was repeated five times for each sample, and the final result is presented as an average. Samples of 50 ± 1 mg were used to determine the ignition/explosion temperature. The measurement was carried out in the range of 100–400 °C, with a heating rate of 5 K/min.

### 3.7. Determination of Linear Combustion Velocity

**Figure 1:**
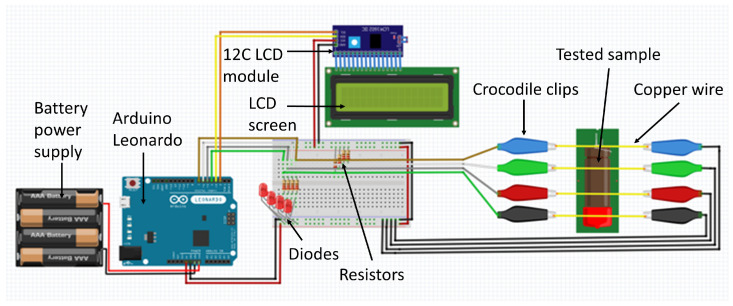
Schematic diagram of the experimental set-up for measuring linear combustion velocity.

Electrical resistance measurement methods were used to determine the linear combustion velocity. The utilised experimental set-up (Figure 1) measured the time between state changes (conducting/non-conducting) at the inputs. These changes were caused by the moving flame front and the burning through of successive wires attached to the sample. The measuring device used gave the time in which the flame front covered the distance with millisecond accuracy.

Samples for the determination of linear combustion velocity were placed in a cellulose tube, measuring h = 9.7 cm, ϕ = 1.0 cm. The determination of linear combustion velocity was repeated five times for each sample, and the final result is presented as an average. The bulk density for each sample was 1.2 ± 0.1 g/cm^3^.

### 3.8. Study of the Kinetics of High-Energy Transformations

To analyse the thermal exothermic decomposition processes of the rocket propellant samples, differential scanning calorimetry (DSC) was used. Measurements were taken using a Mettler Toledo DSC 3 instrument, operating with a maximum temperature range from −90 to 700°. The measurements for each of the samples were performed for approximately 1 ± 0.1 mg of the sample placed in a sealed aluminium vessel. The samples were heated between in a range of 20–450° for different heating rates of 3, 5, 7, 10, 12, and 15 K/min.

The linear relationship between exothermic peak temperature and heating rate can be used to determine thermokinetic parameters of thermal decomposition (activation energy) according to the Kissinger method. This method has been used to determine the activation energy for the decomposition of the investigated samples, being used analogously as in the literature [33,34].

## 4. Conclusions

The obtained results show a significant effect of the utilised binder and oxidising agent on the properties of solid rocket propellants. Interestingly, the combination of an energetic binder with a “green” oxidising agent leads to the lowest observed energetic decomposition activation energy. It should, however, be noted that apart from activation energy, the total heat of combustion of the propellants is also of significant importance in shaping their performance.

As far as the binder comparison is concerned, the use of an energetic binder is more favourable from the point of view of the requirements of solid propellants. The presence of additional energetic groups in the polymer structure (i.e., azide) contributes to their improved properties. Differences can also be seen in the oxidants used. The use of AP leads to higher performance, related to propellant efficiency, compared to PSAN. However, as a result of the formation of toxic gaseous thermal decomposition products of AP, it seems preferable to use more “green” oxidisers, such as PSAN.

The interplay between the choice of binder and oxidising agent is well-illustrated by the fact that replacing GAP with HTPB in propellants containing ammonium perchlorate results in lowering their linear combustion velocity by approximately 36 %. Conversely, the loss of performance associated with replacing AP with the less energetic PSAN (loss of approximately 45 % combustion velocity) can be mostly mitigated by using the energetic binder in place of the traditional one, resulting in only an approximately 12 % loss in combustion velocity (SRP-3 vs. SRP-2). This is a significant factor in favour of transitioning to “green” and high-performance components in the design of solid propellants.

## Figures and Tables

**Table 1:** Friction and impact insensitivity of the produced solid rocket propellant (SRP) samples.

Sample	Friction Sensitivity (N)	Impact Sensitivity (J)
SRP-1 (GAP + AP)	84	5
SRP-2 (HTPB + AP)	120	2
SRP-3 (GAP + PSAN)	112	7.5
SRP-4 (HTPB + PSAN)	160	50

**Table 2:** Determined ignition/explosion temperature of solid propellant samples. The listed temperature is an average of n = 5 experiments.

Sample	SRP-1	SRP-2	SRP-3	SRP-4
**Ignition/explosion temperature (°C)**	320	336	229	259

**Table 3:** Determined linear combustion velocity of samples.

Sample	SRP-1	SRP-2	SRP-3	SRP-4
**Linear combustion velocity (mm/s)**	6.261	3.986	3.488	2.161

**Table 4:** DSC investigation of the utilised binders prior to curing.

	Sample	HTPB	GAP
Property			
**Glass transition (°C)**		−76.3	−55.7
**1st exothermic peak (°C)**		204	246.7
Δ **h_P1_ (J/g)**		245	610
**2nd exothermic peak (°C)**		376	n/a
Δ **h_P2_ (J/g)**		819	n/a

**Table 5:** Kinetics of high-energy transformation of SRP-1.

β ^a^	Peak Temperature (K)	ln (β/T^2^)	1·10^3^/T_p_	Δh (J/g)
3	597	−11.687	1.674	1322
5	594	−11.164	1.683	1751
7	599	−10.845	1.669	1550
10	610	−10.524	1.639	1460
12	611	−10.346	1.636	1531
15	612	−10.125	1.634	2037

^a^ heating rate (K/min).

**Table 6:** Kinetics of high-energy transformation of SRP-2.

β ^a^	Peak Temperature (K)	ln (β/T^2^)	1·10^3^/T_p_	Δh (J/g)
3	608	−11.720	1.646	1905
5	621	−11.253	1.610	2011
7	620	−10.913	1.613	1322
10	625	−10.573	1.600	1311
12	633	−10.416	1.579	2185
15	641	−10.219	1.559	2088

^a^ heating rate (K/min).

**Table 7:** Kinetics of high-energy transformation of SRP-3.

β ^a^	Peak Temperature (K)	ln (β/T^2^)	1·10^3^/T_p_	Δh (J/g)
3	469	−11.202	2.132	648
5	475	−10.718	2.104	752
7	477	−10.389	2.096	724
10	485	−10.065	2.063	634
12	488	−9.896	2.049	688
15	489	−9.677	2.045	760

^a^ heating rate (K/min).

**Table 8:** Activation energy determination results using Kissinger method.

Sample	E_a_ (kJ/mol)
SRP-1	148.5
SRP-2	189.7
SRP-3	129.2

**Table 9:** Materials used in this work.

Chemical (Code)	Purity Grade	Source	Notes
Ammonium perchlorate (AP)	>95%	POCH S.A (Gliwice, Poland)	
Phase-stabilised ammonium nitrate (PSAN)	Produced as per [28]		
Hydroxyl-terminated polybutadiene (HTPB)	>90%	Aldrich Chemicals	$M_{n}$ = 5607 g/mol, dispersity = 2.57
Glycidyl azide polymer (GAP)	Synthesised as per [29], $M_{n}$ = 1478 g/mol, dispersity = 1.2		
Dichloromethane	>95%	PureLand	
N,N′-dimethylformamide	>95%	EUROCHEM BGD	
Sodium azide	>99%	Acros Organics	
Ethane-1,2-diol	>95%	Chempur	
Nitroguanidine (NQ)	Synthesised, as per [30]		
Sulphuric acid	>95%	Chempur	95 wt. % solution
Guanidine nitrate	>95%	Sigma Aldrich USA	
Methylene diphenyl diisocyanate (MDI)	>95%	Sigma Aldrich USA	used as cross-linking agent
Magnesium	>95%	POCH S.A (Gliwice, Poland)	89 μm
Dibutylin dilaureate	>95%	Sigma Aldrich USA	used as a catalyst for cross-linking reaction

**Table 10:** Signal listing for the ^1^H NMR spectra of substances synthesised in this work.

Compound	Chemical Shifts (ppm)
GAP (solvent: CDCl_3_)	**4** (δ 1.18-1.20 -CH_2_-O), (δ1.18-1.20 -CH_2_-); **1** (δ 3.36-3.99 O-CH_2_-); **2** (δ 3.36-3.99 O-CH_2_-); **3** (δ 3.36-3.99 -CH_2_N_3)_
NQ (solvent: DMSO-d_6_)	δ 7.46

**Table 11:** Signal listing for the FT-IR ATR spectra of substances synthesised in this work.

Compound	Wavenumber (cm^−1^)
GAP	ν2928 (C-H); ν2865 (C-H); ν2101 (-N_3_); ν1258 (-N_3_); ν1089 (C-O-C)
Nitroguanidine	ν1038 (C-N); ν1394.5 (NO_2_); ν1631 (-NO_2_); ν3259.5 (N-H); ν3339 (N-H)

**Table 12:** Components of prepared rocket propellant samples.

Components, wt. %	SRP-1	SRP-2	SRP-3	SRP-4
Glycidyl azide polymer	25	-	25	-
Hydroxyl-terminated polybutadiene	-	25	-	25
Ammonium perchlorate	52	52	-	-
Phase-stabilised ammonium nitrate (PSAN) ^a^	-	-	52	52
Mg	18	18	18	18
NQ	2	2	2	2
MDI	3	3	3	3

^a^ PSAN was prepared according to ref. [28] and it consisted of 96 wt.% NH_4_NO_3_, 2 wt.% KNO _3_, 1 wt.% CuO, and 1 wt.% ZnO.

**Table 13:** Measured percentage mass increase of AN and PSAN.

Time, min	Mass Increase, % Initial Mass	
	AN	PSAN
30	0.019	0.014
60	0.030	0.018
90	0.039	0.020
120	0.059	0.025
180	0.069	0.029
210	0.069	0.029
330	0.103	0.033
1440	0.425	0.197

## Data Availability

Data are available from the authors on request.
